# Results of Screening in Schools for Visually Impaired Children

**DOI:** 10.4274/tjo.82246

**Published:** 2017-08-15

**Authors:** Pınar Bingöl Kızıltunç, Aysun İdil, Hüban Atilla, Ayşen Topalkara, Cem Alay

**Affiliations:** 1 Kağızman State Hospital, Ophthalmology Clinic, Kars, Turkey; 2 Ankara University Faculty of Medicine, Department of Ophthalmology, Ankara, Turkey; 3 Cumhuriyet University Faculty of Medicine, Department of Ophthalmology, Sivas, Turkey; 4 Dr. Mustafa Kalemli Tavşanlı State Hospital, Ophthalmology Clinic, Kütahya, Turkey

**Keywords:** Blindness, low vision, low vision aids, visual acuity, visually impaired

## Abstract

**Objectives:**

The aim of this study was to identify the causes of visual impairment in children attending schools for students with visual impairment and to identify children suitable for treatment and rehabilitation.

**Materials and Methods:**

All students were examined in our department by a pediatric ophthalmologist and an ophthalmologist experienced in low vision and visual rehabilitation. The children’s medical histories were recorded. All children underwent ophthalmological examination including visual acuity measurement, anterior segment and dilated fundus evaluation, retinoscopy with cycloplegia, and intraocular pressure measurement. The causes of visual impairment were grouped as avoidable and unavoidable. Children with residual visual acuity better than 20/1250 were included in the low vision rehabilitation programme.

**Results:**

A total of 120 patients were evaluated and 79.2% were legally blind (visual acuity less than 0.05), 18.4% had low vision (visual acuity between 0.05 and 0.3), and 0.8% had normal vision (>0.3). The main causes of visual impairment were retinal dystrophies (24.2%) and retinopathy of prematurity (17.5%). Of all diseases related to visual impairment, 27.6% were avoidable. Improvement in visual acuity was achieved with low vision aids in 57.5% of all patients.

**Conclusion:**

The incidence of visual impairment due to avoidable causes can be decreased by ophthalmic screening. Treatment of these children in the early stages of visual development can improve visual acuity. Even in cases with delayed diagnosis, low vision aids are important for visual and neurobehavioral development, and these programmes may improve quality of life and education in these children.

## INTRODUCTION

The estimated number of blind people around the world is 45 million.^1^ This number is expected to increase to 76 million by 2020.^2^ In 1999, it was estimated that there were 1.4 million blind children and each year 500,000 children are becoming blind.^3^ Most of them have treatable or preventable causes. In 1999, VISION 2020: The Right to Sight initiative was launched by the World Health Organization (WHO; Geneva, Switzerland) with the International Agency for the Prevention of Blindness (London, England).^4,5^ This global movement aims to eliminate avoidable blindness by the year 2020 and avoidable childhood blindness is one arm of this project.

The aim of this study was to identify the profile of children going to schools for students with visual impairment in Ankara, the capital city of Turkey, to determine the causes of low vision and blindness, and to identify children suitable for treatment and rehabilitation.

## MATERIALS AND METHODS

There are two schools in Ankara for visually impaired children, the Gören Eller and Mitat Enç Schools for the Visually Impaired. A total of 120 students attending these schools were examined in a period of 6 months and all of the students were included in the study. Ethics Committee approval was obtained from Ankara University.

A detailed medical history was obtained from the students’ parents. The gestational age and weight, family history of eye diseases, consanguinity, any accompanying neurological diseases, and presence of other sensory disabilities such as deafness and speech disability were recorded.

Visual acuity (VA) was measured with Early Treatment Diabetic Retinopathy Study chart if possible; in younger or uncooperative children, Lea symbols were used, and if VA was not measurable with these methods, light perception, projection, and hand movements were tested and noted. The anterior segment was examined using slit-lamp biomicroscopy. Posterior segment examination with indirect ophthalmoscopy and retinoscopy were performed after cycloplegia and dilatation of pupil with 1% cyclopentolate. Intraocular pressure was measured with Tonopen tonometer.

Before pupil dilatation and cycloplegia, low vision aids were tried in all students who had VA more than 20/1250. Electro-optical and telescopic systems were used to evaluate near and distance visual acuities. Colored filters were also tested to reduce light sensitivity and enhance contrast sensitivity.

Visual loss was classified according to the 2010 WHO definition of visual impairment (Table 1).^6^ Blindness and low vision were defined as visual impairment.

The causes of visual impairment were classified according to whether the loss of visual ability was due to avoidable reasons. Prenatal/perinatal infections, trauma, retinopathy of prematurity (ROP), congenital glaucoma, congenital cataract, uveitis, and refractive errors were accepted as avoidable causes. Other diseases such as retinal/corneal dystrophies, congenital eye anomalies, and cortical blindness were grouped as unavoidable causes.

## RESULTS

A total of 120 children were included in the study. Sixty-nine (57.5%) of them were male and 51 (42.5%) were female and the mean age was 11.5±2.84 years. The ages of the youngest and oldest children attending these schools were 6 and 20 years respectively.

Of all 120 patients, 95 (79.2%) were legally blind, 22 (18.4%) had low vision, and 1 (0.8%) had normal vision. VA could not be assessed in 2 patients (1.6%) due to mental retardation (Table 2). Of the 95 legally blind patients, 69 (72.6%) had only light perception.

The etiological classification of visual impairment is shown in Table 3. The main causes were retinal dystrophies and ROP, with 29 (24.2%) and 21 (17.5%) patients, respectively. A history of consanguinity was present in 48 patients (40%). Among these, the most common disease was retinal dystrophies (19 patients, 39.6%). The other common diseases were congenital eye anomalies and congenital glaucoma, with 7 (14.6%) and 6 (12.5%) patients, respectively. In addition, 24 patients (20%) had family history of visual impairment. The diagnoses of patients with family history and consanguinity were shown in Table 3.

Nineteen (15.8%) patients had associated neurological diseases. Fourteen (73.7%) had epilepsy, 2 (10.5%) mental retardation, 2 (10.5%) cerebral palsy, and 1 (5.3%) had craniofacial anomalies.

Deafness and speech disorders were other sensory disabilities accompanying visual impairment; 6 patients (5%) had speech disorders and 2 (1.7%) were also deaf.

Of all diseases related to visual impairment, 27.6% were avoidable whereas 72.4% were unavoidable. Forty-three patients (35.8%) were using spectacles before examination. Glasses were prescribed to an additional 28 patients after examination. Four patients (3.3%) were scheduled for surgery. These operations were keratoplasty (2 patients), combined keratoplasty and lens extraction (1 patient) and strabismus surgery (1 patient); however, only 1 patient consented to the surgery. Bilateral keratoplasty was performed with the diagnosis of corneal dystrophy. In both eyes, VA was counting fingers before the surgery and increased to 0.1 (Snellen) after the surgery.

The visual acuities of 69 patients (57.5%) increased with low vision aids. Low vision aids used for near and far distances were electro-optical and telescopic systems. Of all 120 patients, 26 (21.7%) had an increase in VA with only electro-optical systems, while 43 (35.8%) used both electro-optical and telescopic systems. The types of telescopic systems used are shown in Tables 4 and 5.

Eight patients (6.6%) had improvement in VA with infrared and/or ultraviolet filter glasses (6 with albinism, 1 dyschromatopsia, 1 rod-cone dystrophy).

## DISCUSSION

Childhood blindness accounts for about 4% of all blindness.^7^ Scoring systems like the disability-adjusted life year can estimate the lifelong burden of a disease. Although childhood blindness seems to be rarer than adult blindness, it results in a similar or higher disability score than adult blindness, so prevention programs for childhood blindness and early diagnosis/treatment of these children are crucial for quality of life and improving visual and neurobehavioral development.

The WHO uses both an anatomical and etiological classification system for causes of childhood blindness^8^ and these causes are also grouped as preventable, treatable, unpreventable and untreatable causes. The major preventable causes of childhood blindness are vitamin A deficiency, measles, ophthalmia neonatorum, and the harmful use of traditional eye care methods. The major treatable causes are cataract, ROP, and glaucoma.^9^

Both the prevalence and causes of childhood blindness vary according to the socioeconomic development of the countries. Three-quarters of blind children live in the poorest countries such as Africa and Asia.^10^ The prevalences of childhood blindness in developed and developing countries are 0.3/1000 and 1.5/1000, respectively.^11^ The main causes of childhood blindness are different in developed and undeveloped countries. While genetic and hereditary diseases seem to be the most frequent causes in developed countries, infectious/contagious diseases and nutritional deficiencies are the most common causes in undeveloped countries.^12^

According to the etiological classification, the main causes are corneal diseases and cataract in undeveloped countries, ROP in developing countries, neurological diseases in developed countries.^13^

Santos-Bueso et al.^12^ evaluated the main causes of childhood blindness in a developed (Morocco) and an undeveloped (Ethiopia) country. Hereditary pathologies and refractive errors were the main causes in the Moroccan population, while corneal diseases and trauma were predominant in the Ethiopian population. Heijthuijsen et al.^14^ showed that the main anatomical site of severe visual impairment and blindness was the retina (in 33.8% of cases) in the Republic of Suriname, a middle-income country.

One of the important avoidable causes of childhood blindness in developing countries is pediatric cataract. After controlling measles and vitamin A deficiency in developing countries, the number of childhood blindness due to cataract increased. The rate of lens blindness in different regions is estimated as 22% in Africa, 5.8% in the Americas, 13.2% in the Eastern Mediterranean, 15.2% in Europe, 13.6% in Southeast Asia, and 21.3% in the Western Pacific.^15^ Aghaji et al.^16^ evaluated 124 children with severe visual impairment and blindness in Southeast Nigeria. They found that the lens was the most common anatomical site of blindness (33.9%), 38.6% of cases were treatable, and 73.4% of all blindness was due to avoidable causes.

In the United Kingdom, the most common diseases were cortical visual impairment, retinal disorders such as ROP, and optic nerve disorders, with rates of 48%, 29%, and 28%, respectively.^17^ Similarly, Kong et al.^15^ determined that the 3 leading causes of childhood blindness in the United States were cortical visual impairment, optic nerve hypoplasia, and ROP, with rates of 18%, 15%, and 14%, respectively.

Various studies have described the results of childhood blindness in Europe.^18,19,20^ In developed European countries, the leading causes were lesions of the central nervous system, congenital anomalies, and retinal disorders. In the middle-income countries of Europe, these causes were congenital cataract, glaucoma, and ROP.

There are many studies evaluating children with low vision and blindness in Turkey. Cetin et al.^21^ found the rate of avoidable and preventable causes of childhood blindness as 69.4%. In contrast, other studies reported lower frequencies of preventable causes.^22,23,24^ The main causes identified by Özen Tunay et al.^23^ were hereditary macular dystrophy and cortical blindness, while Idil^22^ determined the main causes to be hereditary macular degenerations, albinism, and optic atrophy, and hereditary pathologies were shown as the main causes of childhood blindness by Turan et al.^24^ Our results were similar to these studies; the main causes were retinal dystrophies (24.2%), ROP (17.5%), and congenital eye anomalies (14.2%) in our study. Avoidable causes accounted for 27.6% of all cases. These findings are consistent with results in developed countries. Neonatal and early childhood ophthalmic screening tests performed by pediatricians, family practitioners, or ophthalmologists will help to diagnose avoidable diseases earlier, and early treatment will decrease the number of visually impaired children. In children with visual impairment due to delayed treatment or untreatable diseases, visual rehabilitation with low vision aids will increase visual acuity, and this improvement in vision will facilitate the education of these children.

Another important problem in Turkey is consanguinity; retinal dystrophies and congenital eye anomalies are more common in the Turkish population due to high consanguinity rates. The rate of consanguinity in blind children was found as high as 40%. Increasing public awareness about consanguinity may help to decrease the incidence of these diseases.

### Study Limitations

The main limitation of this study is the study population. In this study, we only evaluated the children going to the target schools. Most children with low vision or blindness do not have the chance to go to these schools, so this is not a community-based study. For this reason, more extensive studies involving children who cannot attend school should be undertaken.

## CONCLUSION

In our study population, most of the children achieved significant improvements in vision with rehabilitation, so attempting visual rehabilitation with low vision aids may give some of these children an opportunity to receive education with their peers without social isolation.

## Figures and Tables

**Table 1:**
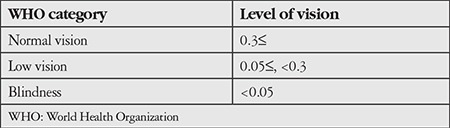
Classification of visual impairment according to the 2010 World Health Organization definition

**Table 2:**
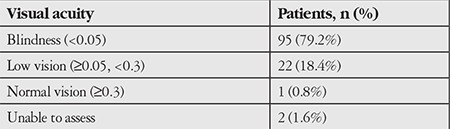
Distribution of the patients according to their visual acuities

**Table 3:**
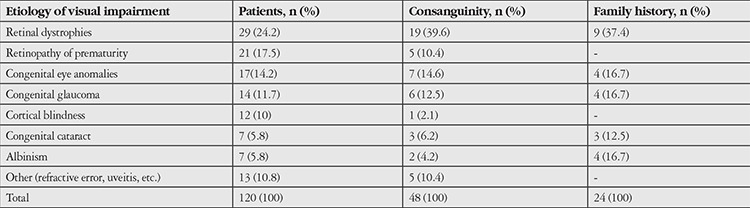
Causes of visual loss, numbers of consanguinity and family history in students attending to the school for students with visual impairment, in Ankara

**Table 4:**
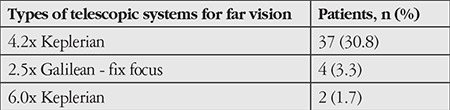
Telescopic systems used for far vision

**Table 5:**
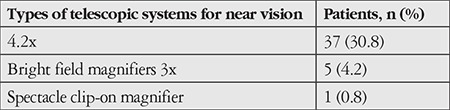
Telescopic systems used for near vision
